# Photostriction of strontium ruthenate

**DOI:** 10.1038/ncomms15108

**Published:** 2017-04-24

**Authors:** Tzu-Chiao Wei, Hsin-Ping Wang, Heng-Jui Liu, Dung-Sheng Tsai, Jr-Jian Ke, Chung-Lun Wu, Yu-Peng Yin, Qian Zhan, Gong-Ru Lin, Ying-Hao Chu, Jr-Hau He

**Affiliations:** 1 Computer, Electrical and Mathematical Sciences and Engineering, King Abdullah University of Science and Technology, Thuwal 23955-6900, Kingdom of Saudi Arabia; 2 Graduate Institute of Photonics and Optoelectronics, National Taiwan University, Taipei 10617, Taiwan; 3 Department of Materials Science and Engineering, National Chung Hsing University, Taichung 4022, Taiwan; 4 School of Materials Science and Engineering, University of Science and Technology Beijing, Beijing 100083, China; 5 Department of Materials Science and Engineering, National Chiao Tung University, Hsinchu 30010, Taiwan; 6 Institute of Physics, Academia Sinica, Taipei 15529, Taiwan

## Abstract

Transition metal oxides with a perovskite crystal structure exhibit a variety of physical properties associated with the lattice. Among these materials, strontium ruthenate (SrRuO_3_) displays unusually strong coupling of charge, spin and lattice degrees of freedom that can give rise to the photostriction, that is, changes in the dimensions of material due to the absorption of light. In this study, we observe a photon-induced strain as high as 1.12% in single domain SrRuO_3_, which we attribute to a nonequilibrium of phonons that are a result of the strong interaction between the crystalline lattice and electrons excited by light. In addition, these light-induced changes in the SrRuO_3_ lattice affect its electrical resistance. The observation of both photostriction and photoresistance in SrRuO_3_ suggests the possibility of utilizing the mechanical and optical functionalities of the material for next-generation optoelectronics, such as remote switches, light-controlled elastic micromotors, microactuators and other optomechanical systems.

## Abstract (web summary)

Light-induced deformation known as photostriction could be used for green energy devices but in most materials the effect is too small to be of practical use. Here, Wei *et al*. study the photostriction of strontium ruthenate and find photon-induced strain efficiencies of more than one percent.

In recent decades, developing clean energy technology has become a major scientific challenge. As we aim to relieve our dependence on fossil fuels, scientists have studied new ways of generating electrical energy, such as solar cells^1^, fuel cells^2^, nanogenerators^3^ and more. However, another approach to green technology is the development of novel materials that use clean sources of energy directly to produce a physical response, thus bypassing the need to generate electricity altogether. One example of this is photostriction, the property in which a material directly converts light into mechanical energy. This generation of strain can then be harnessed for wireless remote control applications, such as photostrictive actuators^4^,^5^, energy harvesting devices^6^ and other optomechanical sensors^7^.

Many materials show photostrictive behaviour, including ferroelectrics^8^,^9^,^10^, organic polymers^11^,^12^ and inorganic semiconductors^13^,^14^,^15^. However, the mechanisms of the photostrictive effect vary depending on the material and are often poorly understood, thus limiting practical applications. For example, light-induced deformation in nonpolar semiconductors, such as crystalline germanium and silicon, is due to a slight change in the atomic bond length induced by excess excited carriers in the conduction band. However, the resulting strain is very small (0.08% and 0.09% for germanium and silicon, respectively)^16^. In organic polymer systems, larger strain is obtained through photo-induced molecular reorientation or ionization reactions^17^. However, these materials are often unstable in air or at high temperatures. Finally, the photostrictive effect in most ferroelectric materials occurs through a combination of the photovoltaic effect and the converse piezoelectric effect, yet it can only be observed below the Curie temperature^18^,^19^ or in nanoscale structures^20^. Kundys *et al*.^21^ measured a substantial visible-light-induced size change in ferroelectric BiFeO_3_ (BFO) crystals using a capacitance dilatometer that has to date displayed the fastest photostrictive response (ps to ns)^17^.

In our search for new photostrictive materials, we decided to explore the transition metal oxide with perovskite structure, SrRuO_3_ (SRO). SRO has received increasing attention due to its high absorption across the visible spectrum^22^ and strong coupling of charge, spin and lattice degrees of freedom at room temperature^23^. The absorption coefficient of SRO at 532 nm is ∼2 × 10^5^ cm^−1^, and this is 25 times higher than silicon and one order of magnitude greater than BFO^23^,^24^. In addition, transition metal oxides are among the most interesting materials due to their unique blend of multiple physical properties, including superconductivity^25^,^26^, piezoelectricity^27^,^28^,^29^, ferroelectricity^30^,^31^, semiconductivity^32^ and thermoelectricity^33^,^34^.

The deformation or rotation of the corner-sharing octahedral oxygen (for example, BO_6_) in perovskite structures plays an important role in determining the magnetic and electronic properties^35^,^36^,^37^ of the material. The metallic perovskite SRO shows significant hybridization between the O 2*p* and Ru 4*d* orbitals^38^, thus leading to a strong coupling of the lattice degrees of freedom^39^ and electromagnetic properties^40^,^41^. Therefore, we expected that a large physical deformation of SRO could be induced by visible light at room temperature, though this has yet to be explored.

In this study, we use micro-Raman scattering to monitor shifts in the optical phonon *A*_g_ mode of SRO to indicate light-induced deformation of the SRO thin films^42^. We also explore how excitation power and temperature affected photostriction in single- and multi-domain SRO structures. In single-domain SRO films at 300 K, we measure the maximum light-induced deformation and photostrictive efficiency to be as high as 1.12% and 7 × 10^−16^ m^3^ W^−1^, respectively. Moreover, we observe that the magnitude of SRO electrical resistance could be controlled with light that also originates from the material's photo-induced deformation behaviour. The strong photostrictive effect of SRO at room temperature offers an attractive route for future devices that can be controlled by means of light alone.

## Results

### Characterization of sample

We began the study by growing 40 nm thick (001)-oriented SRO thin films on a pseudocubic (001) SrTiO_3_ (STO) substrate using pulsed laser deposition (see Methods for more details). A cross-sectional high-resolution transmission electron microscopy (HR-TEM) image of the SRO/STO sample is shown in Supplementary Fig. 1, demonstrating that the SRO features superior crystallinity and a clear lattice structure. The SRO/STO sample features a sharp interface without observable interdiffusion of the species across the boundary.

### Raman selection rules

**Figure 1:**
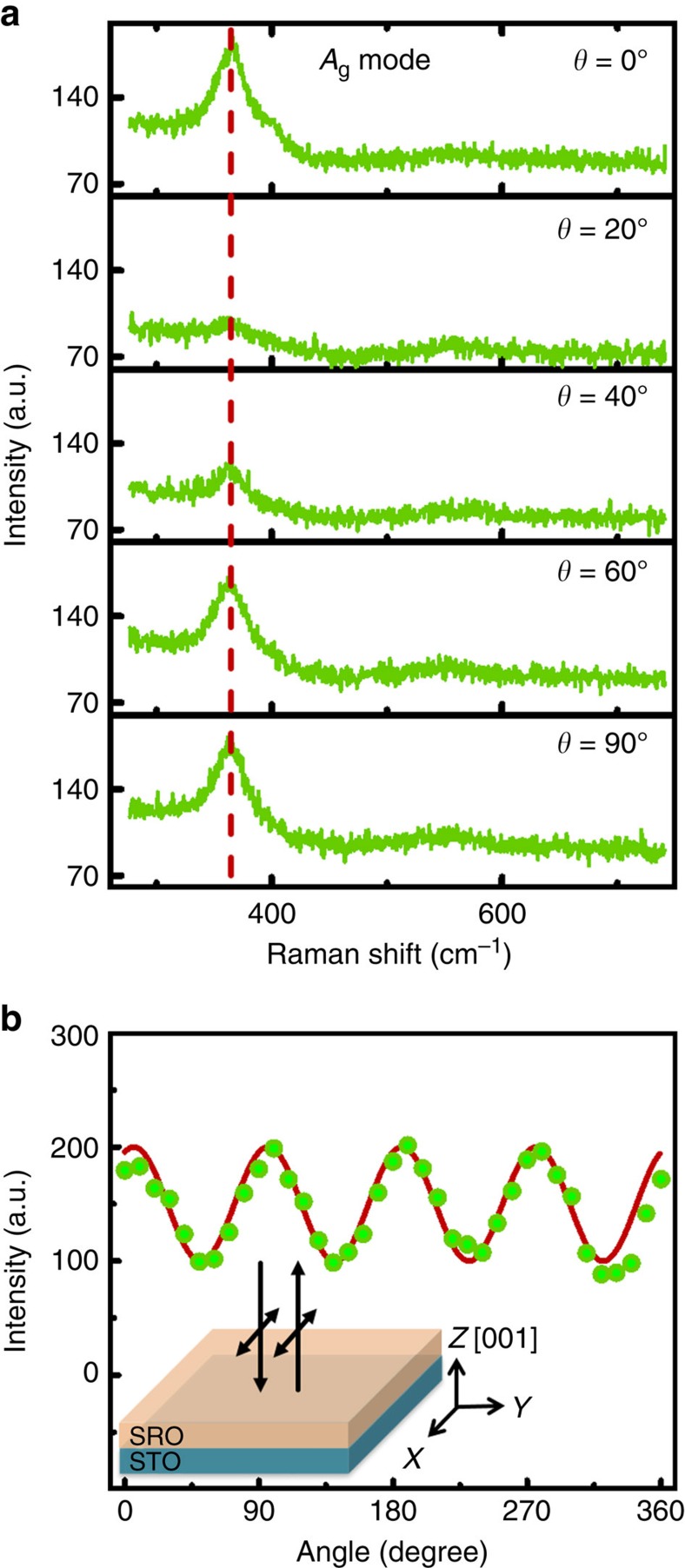
Polarization-dependent Raman scattering. (**a**) Raman spectra of the *A*_g_ phonon mode recorded at the rotation angle, *θ*, of an (001)-oriented SrRuO_3_ thin film epitaxially grown on an (001) SrTiO_3_ substrate. The vertical dashed lines indicate the positions of the *A*_g_ phonon modes of SrRuO_3_. (**b**) The rotation angle dependence of the intensity of the *A*_g_ phonon mode at 372 cm^−1^. The solid line represents the best fit to the calculated intensity of the *A*_g_ phonon mode. The inset shows the normal backscattering geometry used in the polarized Raman scattering system. The arrow represents the propagation and polarization directions of the incident and scattered light, respectively.

At room temperature, SRO has an orthorhombic perovskite structure with the space group *Pnma*^43^. According to nuclear site group analysis, there are 24 Raman active zone-centre optical phonon modes in SRO (7*A*_g_, 5*B*_1g_, 7*B*_2g_ and 5*B*_3g_)^44^. We first examined SRO thin films using Raman spectroscopy, bearing in mind the Raman selection rules. Fig. 1a shows the polarized Raman spectra of (001)-oriented SRO thin films on the STO substrate, employing a normal-incidence backscattering geometry. The sample was rotated around the perpendicular axis with a rotation angle, *θ*. We assigned the intense peak at 372 cm^−1^ to the *A*_g_ symmetry mode (an in-plane vibrational mode of O atoms)^45^. Since the *A*_g_ mode is an in-plane vibrational mode, which shows much higher intensity than other modes, we analysed the in-plane photostrictive effect of SRO by measuring the shift in this peak. The corresponding Raman tensor and scattering intensity of the *A*_g_ mode are given by:


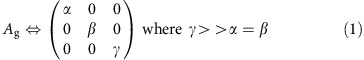



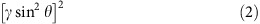


where *α*, *β* and *γ* are the *A*_g_ tensor components along the *x*, *y* and *z* directions, respectively^46^. Considering the expression of the Raman scattering tensor, the *A*_g_ mode can be detected in both *Z*(*XX*)*Z* and *Z*(*YY*)*Z* configurations due to the symmetry properties of the material, in which *X*,*Y* and *Z* represent the lattice directions *a*,*b* and *c*, respectively. Using *Z*(*XX*)*Z* and *Z*(*YY*)*Z*, the two letters on both sides of the brackets represent the propagation direction of the incident and scattered light, respectively. The two letters inside the parentheses represent the polarizations of incident and scattered light, respectively. According to Raman selection rules, the intensity of the *A*_g_ mode of monocrystalline SRO is periodic with the polarization direction of the incident light. The intensity of the *A*_g_ mode as a function of *θ* is shown in Fig. 1b. The scattering intensity of the SRO thin films shows a 90° periodicity with a maximum amplitude at *θ*=10°,100°,190° and 280°, demonstrating that our SRO thin film sample was monocrystalline^47^.

### Light-induced deformation of SRO

**Figure 2:**
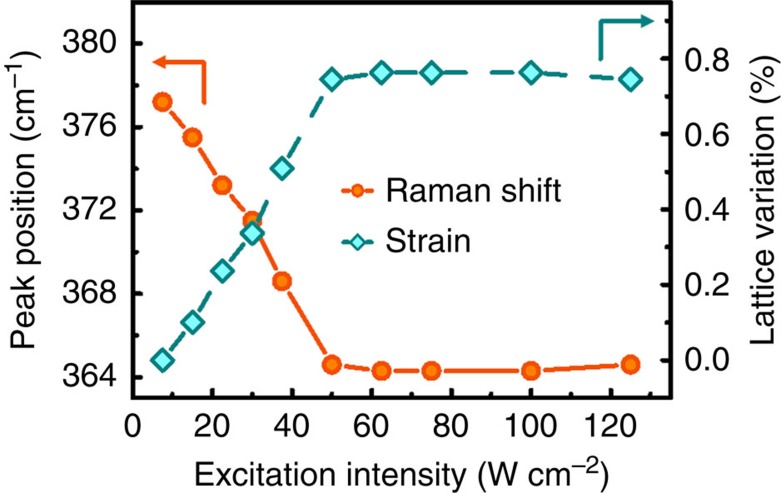
Photon-induced strain measurement of SrRuO_3_/SrTiO_3_. The change in Raman frequency of the *A*_g_ phonon modes and the in-plane lattice variations are shown as a function of excitation intensity.

The generation of a reversible mechanical deformation in materials by the irradiation of light can be estimated from shifts in the Raman spectra, as the vibrational frequencies of the phonon modes change causing a Raman peak shift through the interatomic distances change^48^. We chose a polarized potassium titanyl phosphate (KTP) laser with a wavelength of 532 nm to study the photostriction of SRO on the STO substrate under visible-light illumination (Fig. 2). As we monitored shifts in the *A*_g_ mode of the Raman spectra, we were able to estimate the light-induced SRO lattice strain using the following equation:


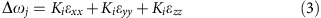


in which Δ*ω*_*j*_ is the phonon frequency shift, *K*_*i*_ is the phonon deformation potential, assuming *K*_*i*_ associated with the stress along the lattice *a*,*b* and *c* axes is the same and equal to 845.93 cm^−1^ for the *A*_g_ mode (372 cm^−1^)^49^,^50^,^51^ and *ɛ*_*xx*_, *ɛ*_*yy*_ and *ɛ*_*zz*_ are the lattice variations along the *a*,*b* and *c* axes, respectively^52^. Because the *A*_g_ mode is independent on the *c* axis, the equation becomes Δ*ω*_*j*_=*K*_*i*_*ɛ*_*xx*_*+K*_*i*_*ɛ*_*yy*_. Then, assuming the lattice variation along the *a* and *b* axes is the same (*ɛ*_*xx*_=*ɛ*_*yy*_), the equation can be further simplified to Δ*ω*_*j*_=2*K*_*i*_*ɛ*_*xx*_ (ref. 53).

**Figure 3:**
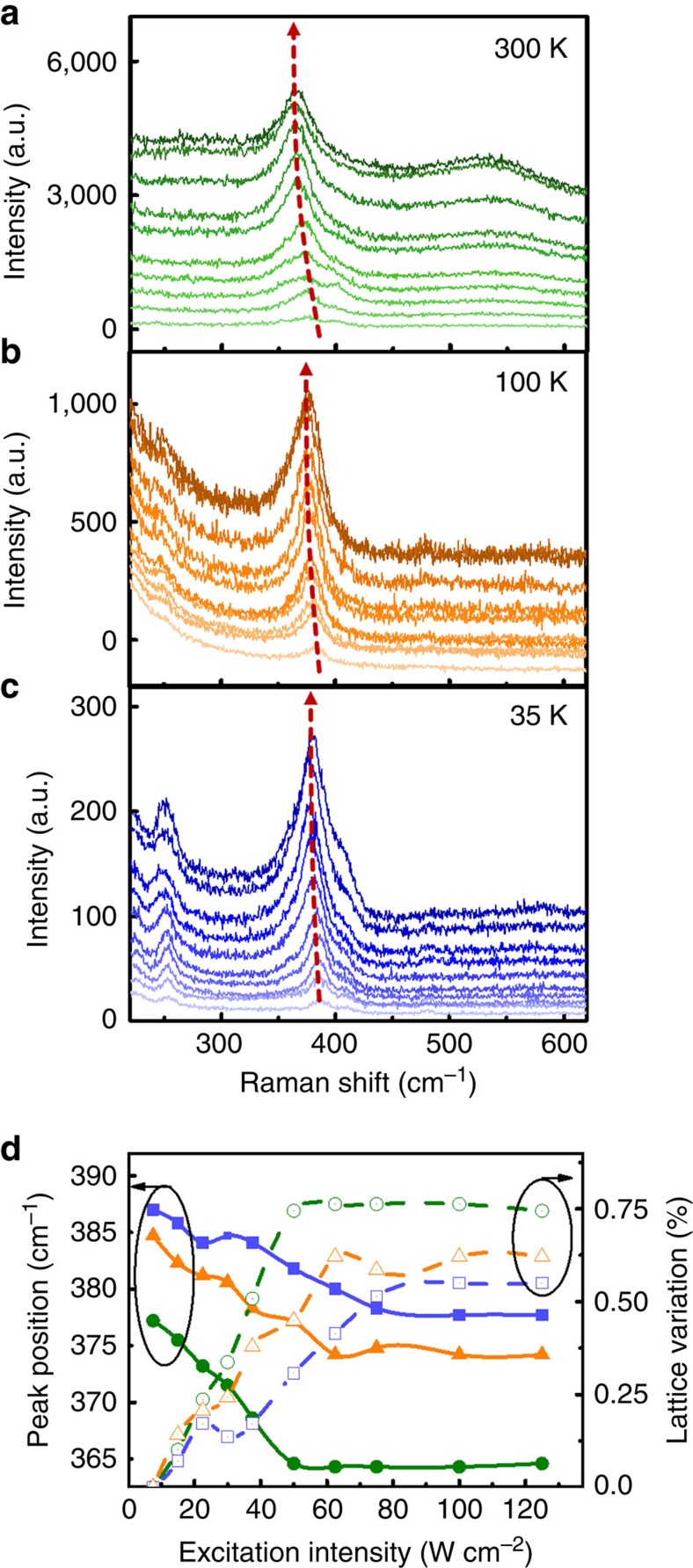
Dependence of photostriction of SrRuO_3_/SrTiO_3_ on temperature and excitation-power intensity. Power-intensity-dependent Raman scattering spectra of SrRuO_3_ thin films at (**a**) 300 K, (**b**) 100 K and (**c**) 35 K, respectively. The arrow indicates the redshift of the *A*_g_ phonon mode induced by the photostrictive effect. (**d**) The Raman frequency of the *A*_g_ phonon mode (filled symbols) and lattice variation (open symbols) as a function of the power intensity at 300 K (circle symbols), 100 K (triangle symbols) and 35 K (square symbols), respectively.

Figure 2 summarizes the excitation power intensity-dependent Raman peak positions of the *A*_g_ mode and the corresponding SRO lattice variation (that is, strain). The Raman scattering spectra appear in the Fig. 3a. The peak position of the *A*_g_ mode exhibited an obvious redshift from 377.2 to 364.6 cm^−1^ with increased laser intensity, implying a significant correlation between excitation power intensity and lattice strain that would suggest SRO is a photostrictive material. After the laser intensity exceeded 50 W cm^−2^, the redshift of the Raman peak and deformation of the SRO thin film remained constant, indicating saturation of photostriction. Based on the previously derived equation (3), the SRO lattice strain reached a maximum value of 0.76%. We observed this phenomenon over several measurements, demonstrating the reversibility of the photostrictive effect in SRO thin films.

To understand the substrate contributions for the photostrictive effect, we also measured the laser intensity-dependent Raman spectra of the STO substrate, as shown in Supplementary Fig. 2. As the 532 nm laser excitation intensity increased from 7.5 to 125 W cm^−2^, there was no obvious shift in the Raman peaks of the STO substrate. Therefore, we can rule out any contribution of the photostrictive effect from this material.

To also exclude laser-induced thermal expansion of the SRO thin film as the reason for the observed *A*_g_ mode peak shift, we also measured the ratio of the Stokes and anti-Stokes Raman scattering intensity to estimate the effective local temperature (*T*_eff_) at the laser focal point (Supplementary Fig. 3a). *T*_eff_ as a function of the incident laser intensity is shown in the Supplementary Fig. 3b. There was no significant change in *T*_eff_ with the laser intensity, demonstrating that we can neglect the laser-induced thermal expansion in the Raman measurements. (see Supplementary Note 1 for more details).

Additionally, the photostrictive effect of SRO saturates at laser intensities of >50 W cm^−2^ that is also inconsistent with light-induced heating effects^11^. To further exclude laser-induced thermal expansion, we also measured the temperature of the SRO thin films using a thermal camera under laser illumination. The noncontact temperature measurements of the SRO samples are shown in Supplementary Fig. 4. We observed a similar change in the surface temperature (2.3 °C increase) under laser illumination. This increase in temperature is too small to result in the large lattice strain on the scale of the photon-induced strain demonstrated in this study (see Supplementary Note 2 for more details).

### Temperature-dependent photostriction of SRO

The temperature-dependent Raman spectra of SRO on the STO substrate using different laser illumination intensities are shown in Fig. 3a-c. The frequency shift, Δ*ω*_*j*_, and the calculated lattice variation of the SRO thin films are summarized in Fig. 3d. It is notable that the light-induced deformation of SRO decreases at lower temperatures. We hypothesize that above the Curie temperature (*T*_C,SRO_ ≈ 144 K), SRO is paramagnetic and essentially has no magnetostriction^23^, which is the effect that occurs when the dimensions of ferromagnetic materials change during the process of magnetization. We believe the light-induced deformation of SRO can be attributed to a phenomenon involving a nonequilibrium of phonons that results from the strong interaction of the lattice and electrons excited by light. Generally, the mechanism of this nonequilibrium of phonons is related to the transfer of energy from light-excited carriers to optical phonons. These excited carriers can induce a large amplitude of lattice motions and then structural deformations by anharmonic couplings via strong electron–electron and electron–phonon interactions^54^. However, below the Curie temperature (< *T*_C,SRO_), SRO becomes ferromagnetic and displays magnetostrictive behaviour. As the laser illuminates SRO thin films, the polarized light changes the distribution of magnetic moments and static magnetization due to the inverse Faraday effect. As a result, such reoriented magnetization by light generates magnetostrictive stress to compress the lattice of SRO due to its negative magnetostriction coefficient^55^ (*η* ∼−2.5 × 10^−3^) that competes with the phonon-mediated lattice expansion. Finally, the superposition of these two mechanisms leads to an insensitive photostrictive effect at lower temperatures (Fig. 3d).

### Lattice domains impact the photostrictive effect

**Figure 4:**
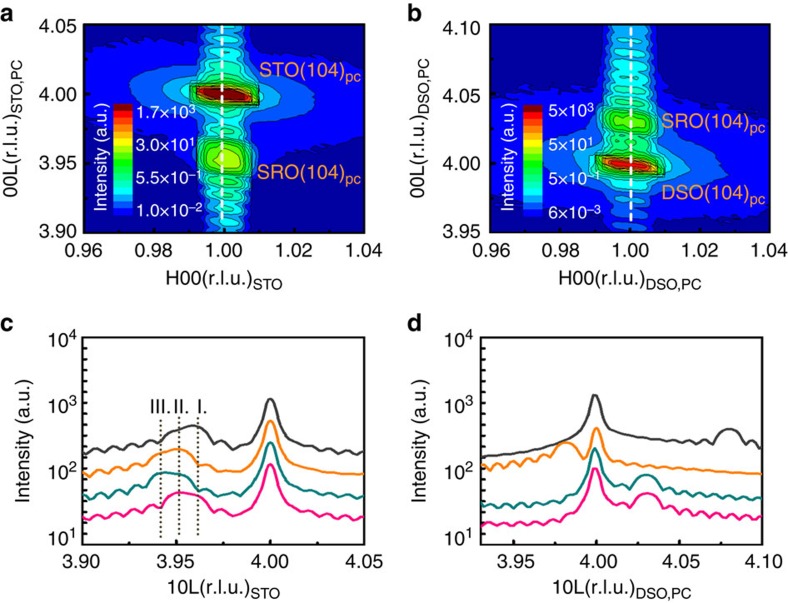
The reciprocal space maps of SrRuO_3_/SrTiO_3_ and SrRuO_3_/DyScO_3_. The reciprocal space maps were taken around the (104) reflection of (**a**) SrTiO_3_ and (**b**) DyScO_3_. The corresponding radial scans along the horizontal vector, **H**=**1** (the vertical dashed lines), with four orthogonal angles, *φ*=0^o^ (black line), 90^o^ (green line), 180^o^ (orange line) and 270^o^ (red line), were collected to inspect the domain structures of SrRuO_3_ thin films grown on (**c**) SrTiO_3_ and (**d**) DyScO_3_ substrates, respectively. In (**c**), the shift in peak due to the tilted angle along the *a* axis of SrRuO_3_ is labelled as I and III. Position II represents diffraction characteristics from the *b* axis of SrRuO_3_.

To further investigate how lattice domains impact the photostrictive effect, we used X-ray diffraction to examine two 40 nm thick (001) SRO thin films deposited on top of (001) STO and (001) DyScO_3_ (DSO) substrates. Figure 4a,b are the reciprocal space maps around the (104) reflection, showing that both the SRO films were coherently grown on the STO and DSO substrates, respectively. Moreover, to carefully inspect the detailed structural information of SRO on these two substrates, we took radial scans along the horizontal vector, **H**=**1**, with four orthogonal azimuthal angles, *φ*=0^o^, 90^o^, 180^o^ and 270^o^ (that is, (104), (014), (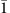
04) and (0
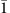
4) reflections of the substrates; Fig. 4c,d).

It is worth noting that all the main peaks of the SRO film on the STO substrate exhibited an asymmetric shape that can be viewed as the convolution of two peaks (Fig. 4c). These deconvoluted peaks can be attributed to the presence of multiple domains in the SRO because the pseudocubic forms a tilted angle along the *a* axis and a rectangular angle along the *b* axis in distorted orthorhombic SRO. This tilted angle causes the peak to shift when the sample is rotated from *φ*=0^o^ to 180^o^, labelled as positions I and III in the radial scans. In addition, position II represents diffraction characteristics from the *b* axis of SRO. Therefore, the two peaks observed in each radial scan shows that the SRO film on the STO substrate possesses two domains. On the other hand, all the main peaks of SRO on the DSO substrate have a symmetric shape, indicating that the SRO film is a single domain (Fig. 4d). Such variation of the lattice domains can be ascribed to the structural difference of the STO and DSO substrates. Usually, there is no preferred growth direction for the tilted SRO lattice structure on the STO substrate since the STO has a cubic structure. However, DSO is also an orthorhombic structure and has the same symmetry group as SRO (*Pbmn*). Hence, the clamping effect of the DSO substrate can easily result in the formation of single-domain SRO thin films.

**Figure 5:**
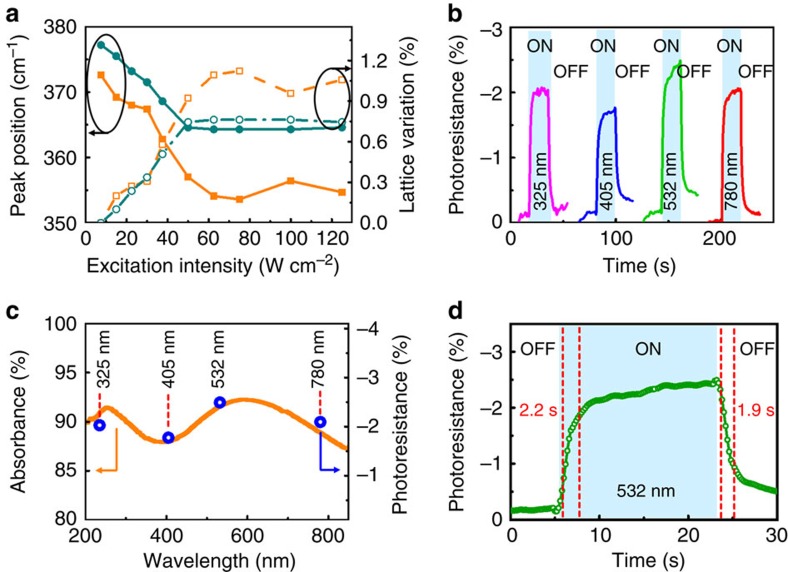
Photostriction and transient photoresistance of SrRuO_3_ thin films. (**a**) The Raman frequency of the *A*_g_ phonon mode (filled symbols) and in-plane lattice variation (open symbols) as a function of excitation power intensity for single- and multi-domain SrRuO_3_ films (that is, grown on DyScO_3_ and SrTiO_3_ substrates, respectively). (**b**) The wavelength dependence of the photoresistive effect measured in SrRuO_3_ thin films on DyScO_3_ substrates as a function of time with fixed bias voltage (2 V) and excitation power intensity (1.0 × 10^5^ W m^−2^). (**c**) The optical absorbance (orange line) and the photoresistance (blue circles) as a function of wavelength for the SrRuO_3_ thin films on DyScO_3_ substrates. (**d**) The transient photoresistance of SrRuO_3_ thin films on DyScO_3_ substrates measured at room temperature under 532 nm laser illumination (excitation power intensity was 1.0 × 10^5^ W m^−2^). The vertical dashed lines indicate the 10 and 90% of the maximum photocurrent.

Interestingly, we observed that the light-induced deformation of SRO thin films was related to these lattice domains. Fig. 5a presents the Raman peak positions of the *A*_g_ phonon mode and the in-plane lattice variation of SRO films on the STO and DSO substrates at different intensities of laser illumination. SRO thin films on the STO (that is, multi-domain SRO) and DSO substrates (that is, single-domain SRO) show different Raman shifts at low excitation intensity that is caused by the different in-plane strain states. As the laser intensity increased, the redshift in the Raman spectra and the expansion of the SRO on the DSO substrate was larger than that of the SRO on the STO substrate. We hypothesize that the counteracting light-induced stress of multi-domain SRO diminishes the total amount of photo-induced deformation of SRO on the STO substrate (0.76%). Meanwhile, the maximum light-induced deformation of the single-domain SRO was measured as high as 1.12% on the DSO substrate. Note that photoexcitation of the substrates did not occur because the energy of the laser (∼2.33 eV) used in the Raman experiments was lower than the bandgap energy of STO (∼3.2 eV) and DSO (∼5.7 eV), and hence there was no possibility of photo-induced strain from these materials.

We can also evaluate the photostrictive behaviour of single- and multi-domain SRO by estimating the photostrictive efficiency at a fixed wavelength using equation (4):


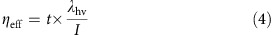


where *t* is the phostrictive material thickness in the direction of illumination, *λ*_hv_ (= Δ*L*/*L*) is the light-induced deformation and *I* is the intensity of the light^17^. As we increased the excitation intensity of the 532 nm laser to 62.5 W cm^−2^, the photostrictive efficiency of single-domain SRO reached a maximum value of 7 × 10^−16^ m^3^ W^−1^ that was much higher than the values for nonpolar semiconductors (3.7 × 10^−20^ m^3^ W^−1^ for cyrstalline germanium and silicon)^16^, and also over 10^8^ times greater than other metal oxides (for example, 5.2 × 10^−26^ m^3^ W^−1^ for PbTiO_3_ and 4 × 10^−25^ m^3^ W^−1^ for BFO)^8^,^56^. We attribute the high photostrictive efficiency of single-domain SRO to its strong optical–structural coupling effects.

### Light-induced photoresistive effect in SRO

As photostriction causes variation in the lattice structure of SRO, it will also induce changes in the electrical resistance of the material. Therefore, in addition to studying light-induced deformation, we also investigated the light-induced photoresistive effect in SRO thin films on DSO substrates. The photoresistive effect can be defined as:


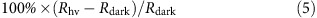


in which *R*_hv_ and *R*_dark_ are the resistances of SRO under illuminated and dark conditions, respectively^57^. We determined the wavelength-dependent photoresistance of SRO using different lasers at various wavelengths (325,405,532 and 780 nm). Note that the measurements of the SRO resistance were taken at room temperature and at a fixed laser intensity of 1.0 × 10^5^ W m^−2^ for all wavelength conditions. The sheet resistance was 18.088 ohm sq^−1^ measured using a standard four-point-probe technique at room temperature. We observed that the change in photoresistance of the samples was fully reversible when the light source was switched between the ON and OFF states (Fig. 5b).

We also compared the photoresistance under different laser wavelengths. As shown in Fig. 5c, the photoresistance of SRO reached its maximum value of ∼2.4% under 532 nm illumination. The spectral absorption of SRO is also shown in Fig. 5c. We attributed the strong optical absorption-dependent photoresistance response of SRO to the illumination-induced excitation of electrons, consistent with previous observations of CoFe films deposited on BFO^58^. This finding implies that optical absorption is an important factor in determining the photoresistance of SRO. Further investigation is required to understand the correlation between the photoconductivity and photostriction of SRO as a function of wavelength.

As shown in Fig. 5d, the transition between the ON and OFF states revealed the response and recovery speeds of photoresistance in the SRO thin films. The rise and fall times under 532 nm illumination (defined as the time between 10 and 90% of the maximum photocurrent) were estimated to be 2.2 and 1.9 s, respectively. There was no obvious variation in the photoresistance response times at different incident wavelengths.

To rule out the effects of temperature on the photoresistance, we also measured the surface temperature of the SRO/DSO sample using a thermometer attached to the SRO film for 90 s during the illumination time period. As shown in Supplementary Fig. 5, the temperature increase caused by the laser was 1.58±0.15 °C. Meanwhile, the time response of the photoresistance of the material was much faster than the change in temperature. Therefore, we feel confident that the photostrictive effect of SRO is not due to thermal expansion by laser illumination.

As compared with the response/recovery speeds of organic polymers^59^,^60^, the SRO thin films demonstrate a fast photoresistance response that can be attributed to the ultrafast dynamics of electrons excited by light and direct electronic-excitation-induced strain in SRO^55^. We expect that the response speed can be further increased by doping^57^ that we are currently investigating.

## Discussion

In summary, we have demonstrated the controlled in-plane photostrictive effect of SRO at room temperature under visible-light illumination. In Raman scattering measurements, the *A*_g_ mode of the SRO thin film showed an increased redshift with laser intensity, indicating a change in the physical dimensions of the material caused by the tensile strain produced by photons. We attribute the underlying mechanism of the observed light-induced deformation to a strong nonequilibrium of phonons in SRO. The excellent light-induced deformation (1.12%) and photostrictive efficiency (7 × 10^−16^ m^3^ W^−1^) generated in single crystallographic domain SRO opens the possibility of utilizing this perovskite in future applications that combine mechanical and optical functionalities. We believe that photostrictive materials like SRO will be the breakthrough needed for the next generation of photostrictive actuators and optomechanical sensors.

## Methods

### Materials

We grew 40 nm thick (001)-oriented SRO thin films on (001) STO and DSO substrates by pulsed laser deposition equipped with *in situ* high-pressure reflection high energy electronic diffraction diagnostics. A KrF excimer laser (*λ*=248 nm) at a repetition rate of 10 Hz and fluence of ∼2.5 J cm^−2^ was used to ablate the SRO target. All the samples were grown at the same substrate temperature of 700 °C and a dynamic oxygen pressure of 100 mTorr. After growth, the samples were cooled to room temperature at a rate of 20 °C min^−1^ in oxygen at 1 atm.

### Raman analysis

To study the photostrictive effects of SRO thin films, we carried out confocal Raman analysis using a Jobin-Yvon T64000 triple spectrometer system equipped with a liquid nitrogen cooled CCD (charge-coupled device) detector. The micro-Raman spectra were measured using a backscattering geometry and a polarized KTP laser (532 nm) (Coherent, Inc.) with a spot size of 40 μm^2^. A temperature-controlled stage was used to measure the spectra from 35 to 300 K. We used an optical filter to control the laser intensity from 7.5 to 125 W cm^−2^. The incident and scattered laser light propagated parallel to the *c* axis of SRO.

### X-ray diffraction techniques

Structural details of the samples were confirmed by synchrotron-based X-ray diffraction at beamline BL-13A at the National Synchrotron Radiation Research Center in Hsinchu, Taiwan. The reciprocal space maps and radial scans were recorded in reciprocal lattice units that were normalized to the substrates (1 r.l.u.=2π/(*a*_STO_ or *a*_DSO,pc_)), in which the cell lattice parameters *a*_STO_ and *a*_DSO,pc_ are 3.905 Å in the cubic form of STO and 3.950 Å in the pseudocubic form of DSO, respectively.

### Photoresistance measurements

The photoresistance of the SRO thin films at various wavelengths (325,405,532 and 780 nm) was measured with a Keithley 4200-SCS semiconductor characterization system. This irradiation experiment was performed at room temperature using a He-Cd laser (325 nm), a GaN laser (405 nm), a KTP laser (532 nm) and a Tm:YAG laser (785 nm) (Coherent, Inc.). The measurements of the SRO resistance were taken at a fixed laser intensity of 1.0 × 10^5^ W m^−2^ for all wavelength conditions.

### HR-TEM image analysis

The crystallinity and atomic structures at the interfaces of the films were investigated using a JEOL-2010 HR-TEM (JEOL Co., Ltd) featuring a high resolution of 0.194 nm operated at an accelerating voltage of 200 kV. The cross-sectional HR-TEM samples were prepared by a standard method, involving mechanical grinding, pre-thinning and a low-angle ion thinning process (Leica EM RES102, Leica Microsystems, Inc.).

### Noncontact temperature measurements

The surface temperatures of the SRO thin films were measured with an InfReC Thermo Gear G100EXD thermal camera (Nippon Avionics Co., Ltd). The camera has 320 × 240 pixels and can take 30 frames s^−1^. It also has a resolution of 0.04 °C at 30 °C and can measure temperatures between −40 and 1,500 °C with an accuracy of ±2%. We measured the temperature of the SRO thin films under 532 nm laser illumination for 3 min using an excitation intensity that was the same as that for the Raman experiments in this study (125 W cm^−2^).

### Data availability

The data that support the findings of this study are available from the corresponding author on reasonable request.

## Additional information

**How to cite this article:** Wei, T.-C. *et al*. Photostriction of strontium ruthenate. *Nat. Commun.* **8,** 15108 doi: 10.1038/ncomms15108 (2017).

**Publisher's note:** Springer Nature remains neutral with regard to jurisdictional claims in published maps and institutional affiliations.

## Supplementary Material

Supplementary Information Supplementary Figures, Supplementary Notes and Supplementary References

## References

[b1] Wang H. P. . Photon management in nanostructured solar cells. J. Mater. Chem. C 2, 3144–3171 (2014).

[b2] Carrette L., Friedrich K. A. & Stimming U. Fuel cells–fundamentals and applications. Fuel Cells 1, 5–39 (2001). 10.1002/1439-7641(20001215)1:4<162::AID-CPHC162>3.0.CO;2-Z 23696319

[b3] Wang Z. L. Towards self-powered nanosystems: from nanogenerators to nanopiezotronics. Adv. Funct. Mater. 18, 3553–3567 (2008).

[b4] Poosanaas P., Tonooka K. & Uchino K. Photostrictive actuators. Mechatronics 10, 467–487 (2000).

[b5] Sun D. C. & Tong L. Y. Modeling of wireless remote shape control for beams using nonlinear photostrictive actuators. Int. J. Solids Struct. 44, 672–684 (2007).

[b6] Lafont T. . Magnetostrictive–piezoelectric composite structures for energy harvesting. J. Micromech. Microeng. 22, 094009 (2012).

[b7] Datskos P. G. . Chemical detection based on adsorption-induced and photoinduced stresses in microelectromechanical systems devices. J. Vac. Sci. Technol. B 19, 1173–1179 (2001).

[b8] Daranciang D. . Ultrafast photovoltaic response in ferroelectric nanolayers. Phys. Rev. Lett. 108, 087601 (2012). 22463572 10.1103/PhysRevLett.108.087601

[b9] Takagi K. . Ferroelectric and photostrictive properties of fine-grained PLZT ceramics derived from mechanical alloying. J. Am. Ceram. Soc. 87, 1477–1482 (2004).

[b10] Kundys B. . Wavelength dependence of photoinduced deformation in BiFeO_3_. Phys. Rev. B 85, 092301 (2012).

[b11] Finkelmann H., Nishikawa E., Pereira G. G. & Warner M. A. New opto-mechanical effect in solids. Phys. Rev. Lett. 87, 015501 (2001). 11461472 10.1103/PhysRevLett.87.015501

[b12] Yu Y., Nakano M. & Ikeda T. Photomechanics: directed bending of a polymer film by light. Nature 425, 145–145 (2003). 12968169 10.1038/425145a

[b13] Buschert J. R. & Colella R. Photostriction effect in silicon observed by time-resolved x-ray diffraction. Solid State Commun. 80, 419–422 (1991).

[b14] Figielski T. Photostriction effect in germanium. Phys. Status Solidi 1, 306–316 (1961).

[b15] Gauster W. B. & Habing D. H. Electronic volume effect in silicon. Phys. Rev. Lett. 18, 1058–1061 (1967).

[b16] Peng C. Y. . Comprehensive study of the Raman shifts of strained silicon and germanium. J. Appl. Phys. 105, 083537 (2009).

[b17] Kundys B. Photostrictive materials. Appl. Phys. Rev. 2, 011301 (2015).

[b18] Tatsuzak I., Itoh K., Ueda S. & Shindo Y. Strain along *c* axis of SbSI caused by illumination in dc electric field. Phys. Rev. Lett. 17, 198–200 (1966).

[b19] Ogasawara T. . General features of photoinduced spin dynamics in ferromagnetic and ferrimagnetic compounds. Phys. Rev. Lett. 94, 087202 (2005). 15783924 10.1103/PhysRevLett.94.087202

[b20] Shih H. Y. . Size-dependent photoelastic effect in ZnO nanorods. Appl. Phys. Lett. 94, 021908 (2009).

[b21] Kundys B., Viret M., Colson D. & Kundys D. O. Light-induced size changes in BiFeO_3_ crystals. Nat. Mater. 9, 803–805 (2010). 20657588 10.1038/nmat2807

[b22] Lee S., Apgar B. A. & Martin L. W. Strong visible-light absorption and hot-carrier injection in TiO_2_/SrRuO_3_ heterostructures. Adv. Energy Mater. 3, 1084–1090 (2013).

[b23] Klein L. . Perpendicular magnetic anisotropy and strong magneto-optic properties of SrRuO_3_ epitaxial films. Appl. Phys. Lett. 66, 2427–2429 (1995).

[b24] Singh A., Khan Z. R., Vilarinho P. M., Gupta V. & Katiyar R. S. Influence of thickness on optical and structural properties of BiFeO_3_ thin films: PLD grown. Mater. Res. Bull. 49, 531–536 (2014).

[b25] Maeno Y. . Superconductivity in a layered perovskite without copper. Nature 372, 532–534 (1994).

[b26] Cava R. J. . Superconductivity near 30 K without copper: the Ba_0.6_K_0.4_BiO_3_ perovskite. Nature 332, 814–816 (1988).

[b27] Saito Y. . Lead-free piezoceramics. Nature 432, 84–87 (2004). 15516921 10.1038/nature03028

[b28] Guo Y. P., Kakimoto K. & Ohsato H. Phase transitional behavior and piezoelectric properties of (Na_0.5_K_0.5_)NbO_3_–LiNbO_3_ ceramics. Appl. Phys. Lett. 85, 4121–4123 (2004).

[b29] Park K. I. . Piezoelectric BaTiO_3_ thin film nanogenerator on plastic substrates. Nano Lett. 10, 4939–4943 (2010). 21050010 10.1021/nl102959k

[b30] Haertling G. H. Ferroelectric ceramics: history and technology. J. Am. Ceram. Soc. 82, 797–818 (1999).

[b31] Cohen R. E. Origin of ferroelectricity in perovskite oxides. Nature 358, 136–138 (1992).

[b32] Shimizu Y., Fukuyama Y., Narikiyo T., Arai H. & Seiyama T. Perovskite-type oxides having semiconductivity as oxygen sensors. Chem. Lett. 14, 377–380 (1985).

[b33] Okuda T., Nakanishi K., Miyasaka S. & Tokura Y. Large thermoelectric response of metallic perovskites: Sr_1-x_La_x_TiO_3_(0 ≤ x ≤ 0.1). Phys. Rev. B 63, 113104 (2001).

[b34] Bocher L. . CaMn_1−x_NbxO_3_ (x ≤ 0.08) perovskite-type phases as promising new high-temperature *n*-type thermoelectric materials. Inorg. Chem. 47, 8077–8085 (2008). 18698764 10.1021/ic800463s

[b35] He J., Borisevich A., Kalinin S. V., Pennycook S. J. & Pantelides S. T. Control of octahedral tilts and magnetic properties of perovskite oxide heterostructures by substrate symmetry. Phys. Rev. Lett. 105, 227203 (2010). 21231419 10.1103/PhysRevLett.105.227203

[b36] Vailionis A. . Misfit strain accommodation in epitaxial ABO_3_ perovskites: lattice rotations and lattice modulations. Phys. Rev. B 83, 064101 (2011).

[b37] Lu W. L. . Strain engineering of octahedral rotations and physical properties of SrRuO_3_ films. Sci. Rep. 5, 10245 (2015). 26018639 10.1038/srep10245 PMC4446894

[b38] Zhou W. P. . Electric field manipulation of magnetic and transport properties in SrRuO_3_/Pb(Mg_1/3_Nb_2/3_)O_3_-PbTiO_3_ heterostructure. Sci. Rep. 4, 6991 (2014). 25384967 10.1038/srep06991 PMC4227015

[b39] Liu H. J. . Epitaxial photostriction-magnetostriction coupled self-assembled nanostructures. ACS Nano 6, 6952–6959 (2012). 22746982 10.1021/nn301976p

[b40] Chen C. L. . Epitaxial SrRuO_3_ thin films on (001) SrTiO_3_. Appl. Phys. Lett. 71, 1047–1049 (1997).

[b41] Singh D. J. Electronic and magnetic properties of the 4*d* itinerant ferromagnet SrRuO_3_. J. Appl. Phys. 79, 4818–4820 (1996).

[b42] Sui Z. & Herman I. P. Effect of strain on phonons in Si, Ge, and Si/Ge heterostructures. Phys. Rev. B 48, 17938–17953 (1993). 10.1103/physrevb.48.17938 10008430

[b43] Kennedy B. J., Hunter B. A. & Hester J. R. Synchrotron X ray diffraction reexamination of the sequence of high-temperature phases in SrRuO_3_. Phys. Rev. B 65, 224103 (2002).

[b44] Iliev M. N. . Raman spectroscopy of SrRuO_3_ near the paramagnetic-to-ferromagnetic phase transition. Phys. Rev. B 59, 364–368 (1999).

[b45] Lucazeau G. Effect of pressure and temperature on Raman spectra of solids: anharmonicity. J. Raman Spectrosc. 34, 478–496 (2003).

[b46] Herranz G. . Domain structure of epitaxial SrRuO_3_ thin films. Phys. Rev. B 71, 174411 (2005).

[b47] Sorianello V., Colace L., Nardone M. & Assanto G. Thermally evaporated single-crystal germanium on silicon. Thin Solid Films 519, 8037–8040 (2011).

[b48] Schadler L. S., Giannaris S. C. & Ajayan P. M. Load transfer in carbon nanotube epoxy composites. Appl. Phys. Lett. 73, 3842–3844 (1998).

[b49] Deluca M. & Pezzotti G. First-order transverse phonon deformation potentials of tetragonal perovskites. J. Phys. Chem. A 112, 11165–11171 (2008). 18841950 10.1021/jp805278u

[b50] Kiyama T., Yoshimura K., Kosuge K., Ikeda Y. & Bando Y. Invar effect of SrRuO_3_: itinerant electron magnetism of Ru 4*d* electrons. Phys. Rev. B 54, R756–R759 (1996). 10.1103/physrevb.54.r756 9985427

[b51] Kirillov D. . Phonon anomalies at the magnetic phase transition in SrRuO_3_. Phys. Rev. B 51, 12825–12828 (1995). 10.1103/physrevb.51.12825 9978064

[b52] Liao M. H., Kuo P. S., Jan S. R., Chang S. T. & Liu C. W. Strained Pt Schottky diodes on n-type Si and Ge. Appl. Phys. Lett. 88, 143509 (2006).

[b53] Antonakos A., Palles D., Liarokapis E., Filippi M. & Prellier W. Evaluation of the strains in charge-ordered Pr_1−x_Ca_x_MnO_3_ thin films using Raman spectroscopy. J. Appl. Phys. 104, 063508 (2008).

[b54] Yang D. S., Gedik N. & Zewail A. H. Ultrafast electron crystallography. 1. Nonequilibrium dynamics of nanometer-scale structures. J. Phys. Chem. C 111, 4889–4919 (2007).

[b55] Schmising C. V. . Ultrafast magnetostriction and phonon-mediated stress in a photoexcited ferromagnet. Phys. Rev. B 78, 060404 (2008).

[b56] Schick D. . Localized excited charge carriers generate ultrafast inhomogeneous strain in the multiferroic BiFeO_3_. Phys. Rev. Lett. 112, 097602 (2014). 24655276 10.1103/PhysRevLett.112.097602

[b57] Kundys B. . Light controlled magnetoresistance and magnetic field controlled photoresistance in CoFe film deposited on BiFeO_3_. Appl. Phys. Lett. 100, 262411 (2012).

[b58] Jin Z. M. . Strain modulated transient photostriction in La and Nb codoped multiferroic BiFeO_3_ thin films. Appl. Phys. Lett. 101, 242902 (2012).

[b59] Sánchez-Ferrer A., Merekalov A. & Finkelmann H. Opto-mechanical effect in photoactive nematic side-chain liquid-crystalline elastomers. Macromol. Rapid Commun. 32, 671–678 (2011). 21480426 10.1002/marc.201100005

[b60] Vanderve G. & Prins W. Photomechanical energy conversion in a polymer membrane. Nature 230, 70–72 (1971).4927019

