# Supplementary material for: Photostriction of strontium ruthenate
Source: Nat Commun. 2017 Apr 24;8:15018. doi: 10.1038/ncomms15108 (PMC5413985; doi:10.1038/ncomms15108)
Supplement: Supplementary Information — Supplementary Figures, Supplementary Notes and Supplementary References [file ncomms15108-s1.pdf]

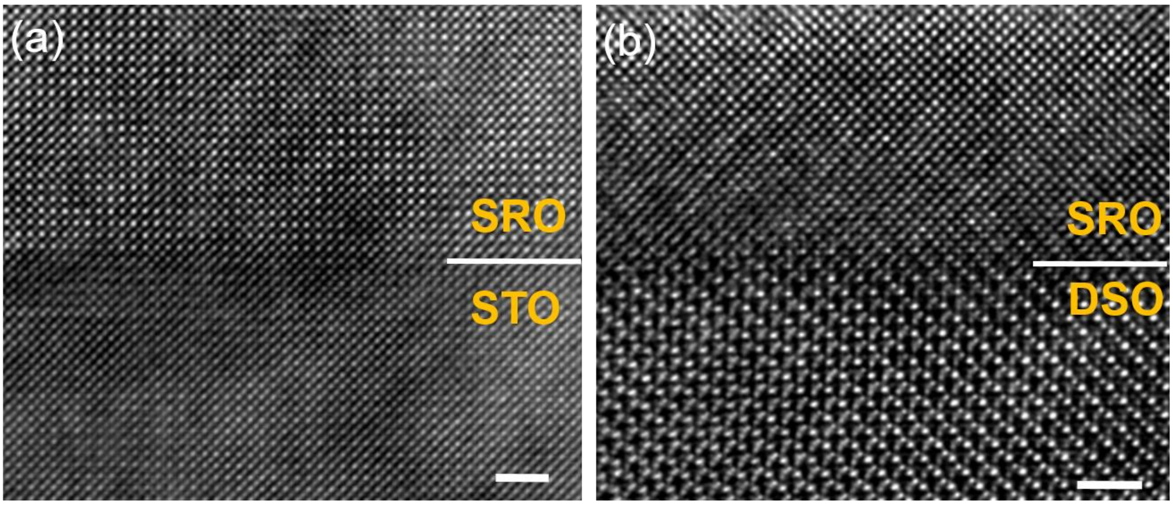

Supplementary Figure 1. Cross-sectional high-resolution transmission electron microscopy (HR-TEM) images. Using HR-TEM imaging, we were able to observe the apparent crystallinity of the (**a**) $SrRuO_3$/$SrTiO_3$ and (**b**) $SrRuO_3$/$DyScO_3$ cross-sectional interfaces (scale bars, 2 nm (**a** and **b**)). We emphasize the location of each interface with the added white line. These images reveal superior crystallinity of $SrRuO_3$ and its clear lattice structure. The $SrRuO_3$/$SrTiO_3$ and $SrRuO_3$/$DyScO_3$ materials feature sharp interfaces without observable interdiffusion of the species across these boundaries. Therefore, we can rule out the possibility that the crystal structure and crystal defects of the $SrRuO_3$ play a role in the observed photostriction.

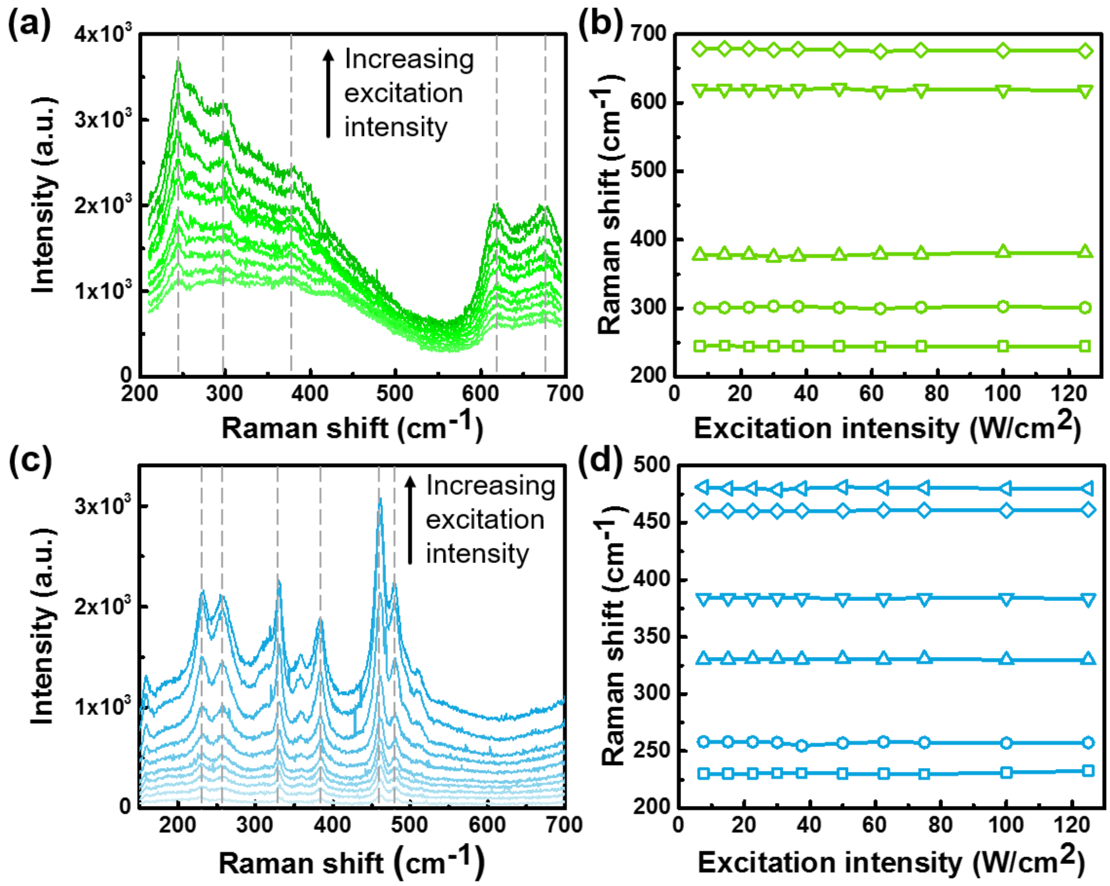

Supplementary Figure 2. Laser intensity-dependent Raman scattering spectra. The 532 nm laser intensity-dependent **(a)** Raman scattering spectra and **(b)** phonon modes of the $SrTiO_3$ substrate. The 532 nm laser intensity-dependent **(c)** Raman scattering spectra and **(d)** phonon modes of the $DyScO_3$ substrate. The vertical dashed lines indicate the positions of the phonon modes of the $SrTiO_3$ and $DyScO_3$ substrates.

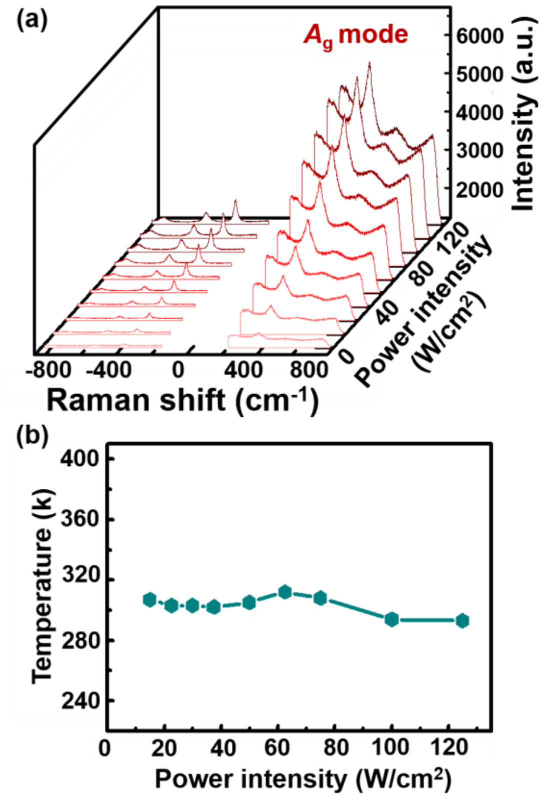

Supplementary Figure 3. Laser intensity-dependent Stokes and anti-Stokes Raman spectra of $SrRuO_3$ thin films. (**a**) The Raman frequency of the A_g phonon mode are shown as a function of excitation power intensity. (**b**) The local temperature at the laser focal point obtained from the Stokes and anti-Stokes intensity ratio (I_S/I_{AS}) as a function of the laser intensity.

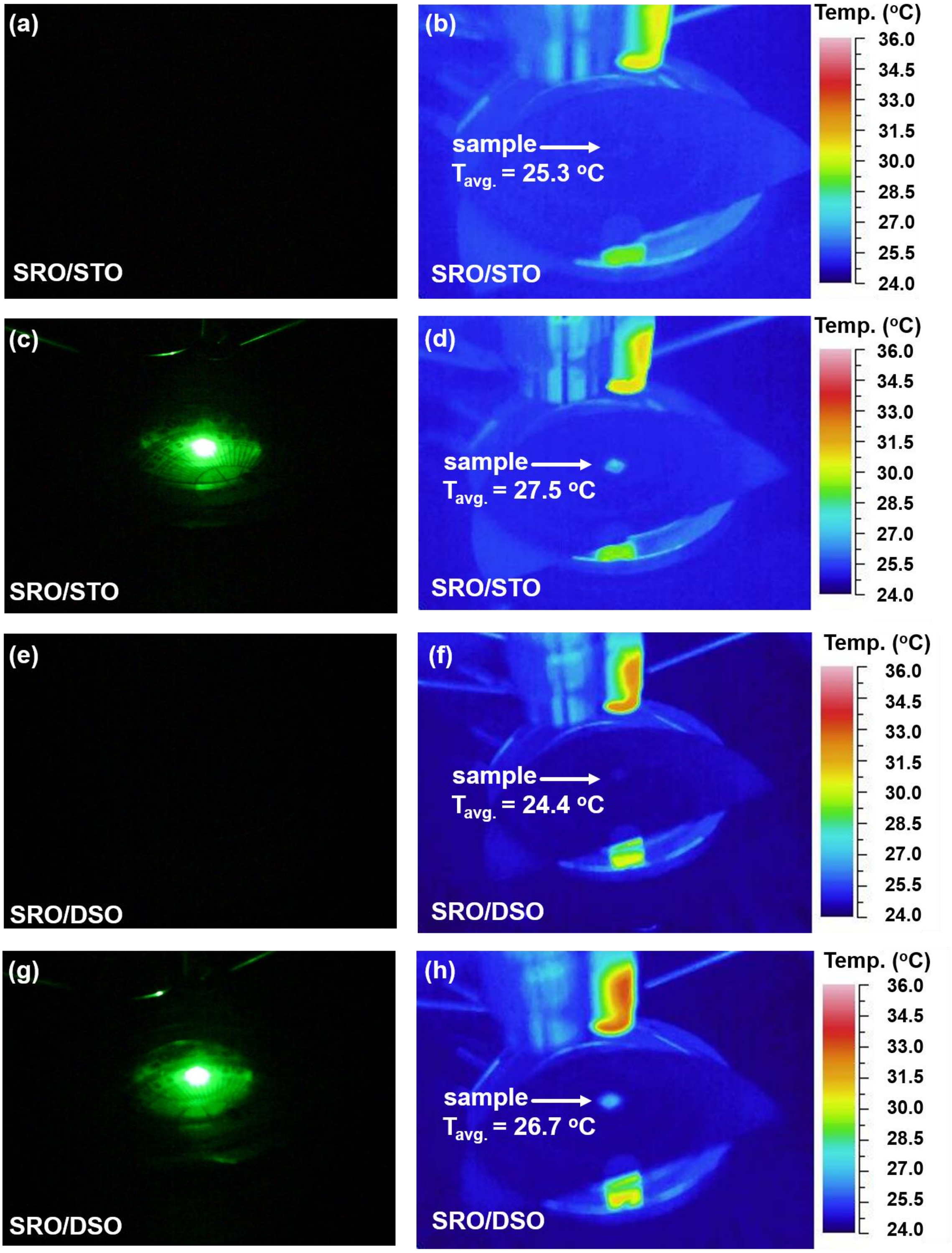

Supplementary Figure 4. Non-contact temperature measurements of the $SrRuO_3$. (a), (**c**) Optical and (**b**), (**d**) thermal images of $SrRuO_3$ on $SrTiO_3$ substrate before and during the laser irradiation, respectively. (**e**), (**g**) Optical and (**f**), (**h**) thermal images of the $SrRuO_3$ thin film on the $DyScO_3$ substrate before and during the laser irradiation, respectively.

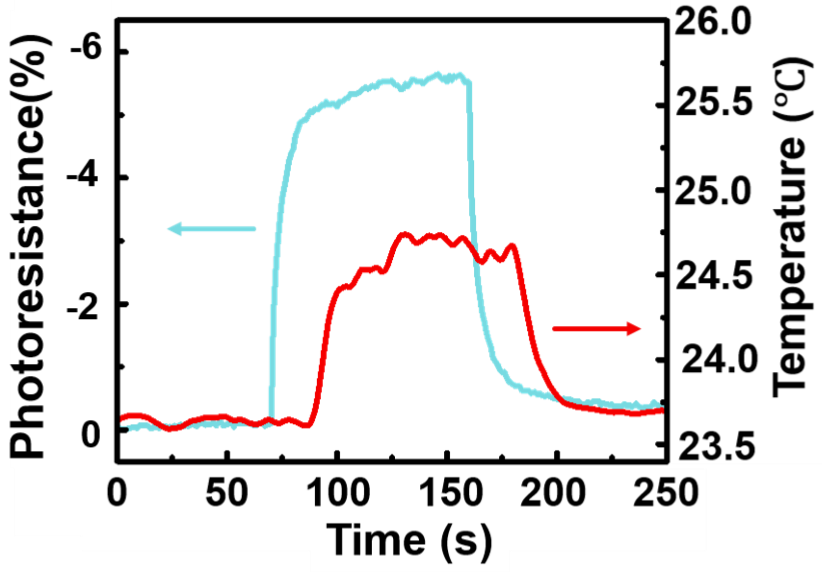

Supplementary Figure 5. The time dependent photoresistance and temperature of the $SrRuO_3/DyScO_3$. The change in photoresistance (left y-axis) and temperature (right y-axis) as a function of time under 532 nm illumination at 50 W cm^{-2}.

Supplementary Note 1: Estimate the effective local temperature at the laser focal point.

Supplementary Figure 3a shows the laser intensity-dependent Stokes and anti-Stokes spectra of the $SrRuO_3$ thin films (the peaks of 372 and -372 cm^{-1}, respectively). The frequency shifts of the Stokes and anti-Stokes scattering were symmetric, but the scattering intensity of the anti-Stokes transition was much lower than the Stokes transition. According to the quantum theory, the local temperature, $T_{\rm eff}$, can be extracted by[1]:

$$T_{eff} = \frac{\frac{\hbar\omega}{k_{\rm B}}}{\ln(\frac{I_{\rm S}}{I_{\rm AS}})} = \frac{1.43859\times\omega}{\ln(\frac{I_{\rm S}}{I_{\rm AS}})} \quad (1)$$

where ω is the Raman frequency, $\hbar$ is the reduced Planck constant, $k_{\rm B}$ is the Boltzmann constant, and $I_{\rm S}$ and $I_{\rm AS}$ are the intensities of the Stokes and anti-Stokes Raman scattering, respectively. $T_{\rm eff}$ as a function of incident laser intensity is shown in Supplementary Figure 3b. There was no significant laser intensity dependance, demonstrating that there was no measurable laser-induced thermal expansion in the material. In addition, the photostriction of SRO depends nonlinearly on the light intensity and saturates beyond 50 W cm^{-2}, which is also inconsistent with the light-induced heating effect[2].

Supplementary Note 2: Measured the temperature of the SRO thin films.

To further exclude laser-induced thermal expansion, we measured the temperature of the $SrRuO_3$ thin films using an InfReC Thermo Gear G100EXD thermal camera (Nippon Avionics Co., Ltd.) under laser illumination for 3 min. The excitation intensity of the laser for the thermal camera measurements was the same as that for the Raman experiments in this study. As shown in Supplementary Figure 3a-d, the temperature in the lab was constant (25.3 ºC), while we observed that the surface temperature of the $SrRuO_3/SrTiO_3$ sample increased by 2.2 ºC (from 25.3 ºC to 27.5 ºC) under laser illumination. Supplementary Figure 3e-h shows the thermal image of the $SrRuO_3/DyScO_3$ sample. We observed a similar change in the surface temperature (2.3 ºC increase) under laser illumination. This increase in temperature is too small to result in a large lattice strain as compared to photon-induced strain demonstrated in this study. Researchers have found that the volume change of $SrRuO_3$ is only about 0.02% from 200 K to 300 K[3]. However, we measured the photon-induced strain of $SrRuO_3$ to be as high as 1.12%.

Supplementary References

1. Balkanski, M., Wallis, R. F. & Haro, E. Anharmonic effects in light scattering due to optical phonons in silicon. *Phys. Rev. B* **28**, 1928–1934 (1983).
2. Daranciang, D. *et al.* Ultrafast photovoltaic response in ferroelectric nanolayers. *Phys. Rev. Lett.* **108**, 087601 (2012).
3. Lee, S. Large in-plane deformation of RuO_6 octahedron and ferromagnetism of bulk $SrRuO_3$. *J. Phys. Condens. Matter* **25**, 465201 (2013).
